# Feasibility of C2 Vertebra Screws Placement in Patient With Occipitalization of Atlas: A Tomographic Study

**DOI:** 10.1097/MD.0000000000001492

**Published:** 2015-09-18

**Authors:** Wei Ji, Xiang Liu, Wenhan Huang, Zucheng Huang, Xueshi Li, Jianting Chen, Zenghui Wu, Qingan Zhu

**Affiliations:** From the Department of Spinal Surgery, Nanfang Hospital, Southern Medical University (WJ, XL, ZH, JC, QZ), and Department of Orthopedics, Guangzhou General Hospital of Guangzhou Military Command (Liuhuaqiao Hospital), Guangzhou, China (XL, WH, ZW).

## Abstract

Occipitalization of atlas (OA) is a congenital disease with the possibility of anomalous bony anatomies and the C2 pedicle screw insertion is technically challenging. However, there are no existing literatures clarified the dimensions and angulations of the C2 pedicles, lamina and lateral masses for screw insertion in patients with OA. Therefore, the aim of this study was to assess the morphometric features of C2 for screw placement in OA to guide the use of surgical screws.

Measurements of the OA patients on the computer tomography (CT) images including lamina angle, length and thickness, pedicle angle, length and thickness, and lateral mass thickness and length of the axis vertebra. The OA patients data were compared with age and gender matched cohort of randomly selected patients in a control group without OA. The picture archiving and communication system was used for all patients who had received cervical CT scanning between January 2001 and January 2015. Measurements were performed independently by 2 experienced observers who reviewed the CT scans and recorded the patients with OA. Statistical analysis was performed at a level of significance *P* < 0.05.

A total of 73 patients (29 males and 44 females) were eligible to be included in the OA group. In most of the measurements the pathological cohort had significantly smaller values compared to the control group (*P* < 0.05). In the OA group, only 45% of the pedicles and 88% of the lamina had thicknesses bigger than 3.5 mm. Both groups had all pedicle and lamina lengths bigger than 12 mm. Regarding the length of the lateral mass, no value was bigger than 12 mm in the OA group, whereas 40% of the values in the control group were bigger than 12 mm. The average pedicle and laminar angles were 37° and 49° in the patients with OA, respectively.

The variable anatomy in patients with OA needs to be taken into account when performing spinal stabilization as the C2 bony architectures are significantly smaller than normal. Anatomically, translaminar screw is a more viable option in comparison with pedicle screw for C2 fixation in OA. Nevertheless, the suitability should be fully assessed prior to surgery.

## INTRODUCTION

Occipitalization of atlas (OA) or atlas assimilation is defined as partial or complete congenital fusion of the occiput and atlas, which is caused by failure of segmentation between the 4th occipital and the 1st cervical sclerotome during embryonic development,^1–3^ and represents approximately one third of all skeletal anomalies of the occipitocervical junction.^4^ Its reported prevalence in the general population has ranged from 0.08% to 2.76%,^5–7^ with males and females being equally affected. OA is always associated with basilar invagination and atlantoaxial dislocation, resulting in compression of the cervicomedullary by the odontoid process.^8^ Neurologic deterioration in such patients commonly presents in the 3rd or 4th decade of life and requires surgical treatment.^9–11^

To successfully stabilize the mechanically compromised occipitocervical junction, one must surgically correct deformity or displacement and decompress neural structures. Occipitocervical fixation spanning from occiput to C2 or atlantoaxial fusion are widely used in disorders related with instability of the occipitocervical junciton.^12,13^ However, in patients with OA, C1 lateral mass, and condyle are hypoplastic and fuse to each other, and the morphology and volume of C1 lateral mass are significantly altered from normal anatomy.^14^ Therefore, C1 lateral mass screw placement is relatively difficult and dangerous.

Fortunately, C2 screws are alternative anchors for occipitocervical fixation and have been successfully used for the treatment of occipitocervical instability.^15–18^ Definitely, OA is a congenital disease with the possibility of anomalous bony anatomies and the data from general population may not apply to patients with OA. However, there are no existing literatures clarified the dimensions and angulations of the C2 pedicles, lamina and lateral masses for screw insertion in patients with OA. Therefore, the aim of this study was to assess the morphometric features of C2 for screw placement in OA and compared with those in patients without OA to guide the use of surgical screws.

## MATERIALS AND METHODS

**FIGURE 1:**
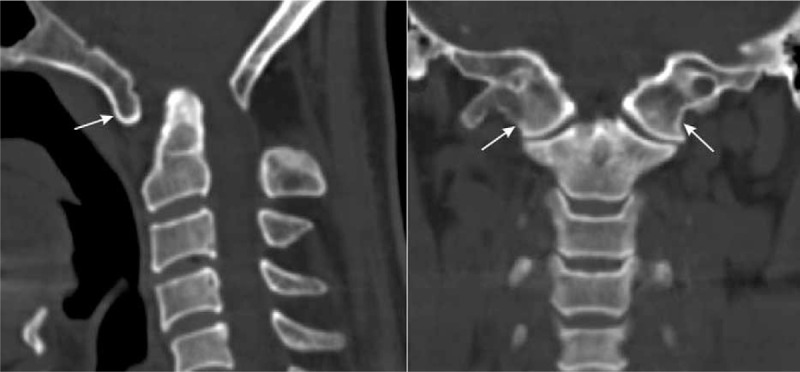
Occipitalization of atlas (arrows) shown in the sagittal plane (left) and the coronal plane (right) on the reconstructed CT images.

This was a retrospective analysis on patients of East Asian ancestry at the Department of Orthopedics in our hospital between January 2001 and January 2015, requiring computer tomography (CT) scanning of the head and cervical spine. Ethical approval and written consents from the participants were waived due to the retrospective design of the present study. However, their personal information were anonymized and deidentified before analysis. Images were initially screened in picture archiving and communication system. Two experienced spinal surgeons reviewed the CT scans of the patients and looked for OA and other congenital malformations of the craniovertebral region such as basilar invagination, Klippel-Feil syndrome, Chiari malformation, and vertebral artery (VA) anomaly. The diagnosis of OA was assessed on sagittal and coronal CT images (Figure 1). CT images were taken using a General Electric CT scanner (Philips Brilliance 16 CT; Philips Medical Systems, Eindhoven, The Netherlands) with slice thickness of 1 mm, pitch of 0.7 mm, 120 kV, 180 mA, 512 × 512 matrix, and reconstruction level of 1 mm. Images of the sagittal and axial planes of the craniovertebral region were obtained after multiplanar reconstruction on the workstation (MXV, Philips).

**FIGURE 2:**
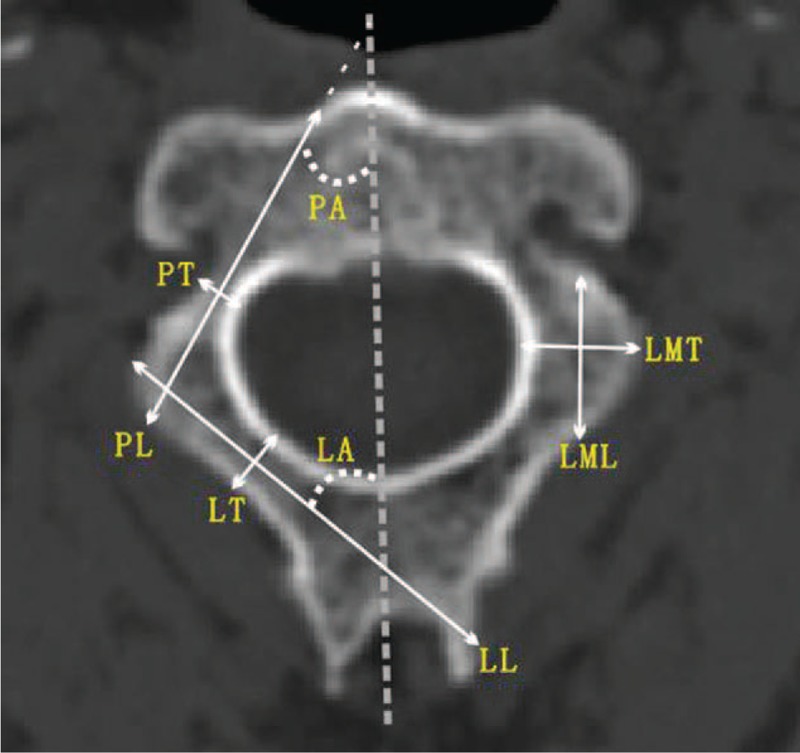
Measurements of C2 on the axial CT image. CT = computer tomography, LA = laminar angle, LL = laminar length, LML = lateral mass length, LMT = lateral mass thickness, LT = laminar thickness, PA = pedicle angle, PL = pedicle length, PT = pedicle thickness.

According to the C2 measurement methods by Cristante et al,^19^ we assessed the dimensions and angulations of right and left laminas, pedicles, and lateral masses in an axial C2 section corresponding to the midpoint of lamina height. C2 pedicle thickness was defined as the narrowest portion of the pedicle (Figure 2). Pedicle length was measured between the entry point in the lateral cortex and the end point in the anterior cortex of C2 body. Pedicle angle was defined as the angle from the axis of pedicle to a line passing through the spinous process and odontoid process, dividing the vertebrae into 2 hemi-vertebrae (Figure 2).

C2 laminar thickness was referred to be the measurement of the narrowest portion of the lamina. In the axial plane, laminar length was measured from the junction of the lamina and spinous process to the contralateral outer cortex of the lateral mass. The laminar angle was measured as the angle from the axis of lamina to a line passing through the spinous process and odontoid process (Figure 2).

The thickness of the lateral mass was measured at its widest point at this same axial section. The length of the lateral mass was measured from the point of transition from the lamina to the mass to the opposite cortex (Figure 2).

A control group without OA was age and gender matched to the OA patient cohort, in which the patients were randomly selected from the same database (random sampling method to analyze measured data on CT scans statistically). Same measurements and procedures as described above were also performed in the control group.

## STATISTICAL ANALYSIS

The statistical analyses were carried out using SPSS (SPSS Inc, Chicago, IL). Data were obtained for mean, standard deviation, and minimum and maximum values. The normality of data distribution was screened with Shapiro-Wilk test. Statistical analyses were performed using either independent-samples *t*-test or paired-samples *t*-test. Statistical significance was set at *P* < 0.05.

## RESULTS

A total of 73 patients (45.0 ± 12.3-years old, mean ± standard deviation, SD) with OA were available for this study. Age ranged between 18 and 73 years, including 29 (39.7%) male and 44 (60.3%) female patients. The control group was age and gender matched to the OA patient cohort (29 males and 44 females). Tables 1 and 2 show the mean values, standard deviations, and minimum and maximum values for the anatomic measurements of the lamina, pedicle, and lateral mass.

**TABLE 1:**
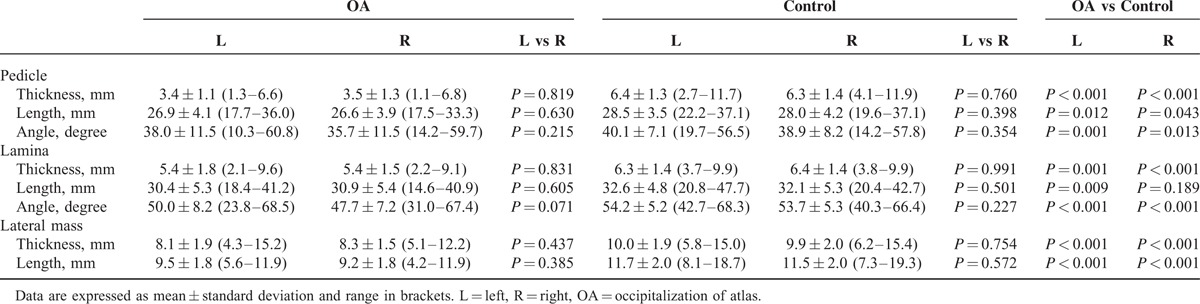
Measurements of C2 in the OA and Control Groups by Laterality

**TABLE 2:**
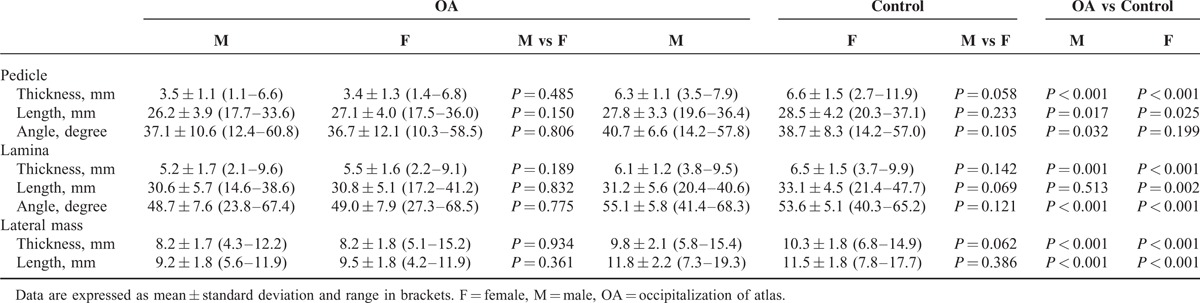
Measurements of C2 in the OA and Control Groups by Gender

In the analysis by body laterality (Table 1), left or right sides in the control group had significantly higher values of measurements than the respective ones in OA group, except the values of laminar length in right side (32.1 vs 30.9, *P* = 0.189). Furthermore, all outcomes revealed no significant differences from the comparison between left and right sides neither in OA group nor in control group. In the analysis by gender (Table 2), females and males in control group had significantly higher values of measurements than that in OA group, except the values of pedicle angle for females (38.7 vs 36.7, *P* = 0.199) and laminar length for males (30.6 vs 31.2, *P* = 0.513). No significant gender differences were found in either of the two groups.

The mean thickness, length, and angle of the C2 pedicle was 3.5 mm, 26.8 mm, and 36.9° in the OA group, respectively, and 6.4 mm, 28.2 mm, and 39.5° in the control group, respectively. In the OA group, 54.8% of the pedicles were observed to have a thickness less than 3.5 mm, whereas only 1.4% patients have thin pedicle in the control group. With regard to the frequency distribution of pedicle thickness, 28.8% of the pedicles were bigger than 4 mm, and 9.6% were bigger than 5 mm. Moreover, 13.7% of the bilateral pedicles were with thickness more than 4 mm, only 1.4% had thickness more than 5 mm (Table 3).

**TABLE 3:**
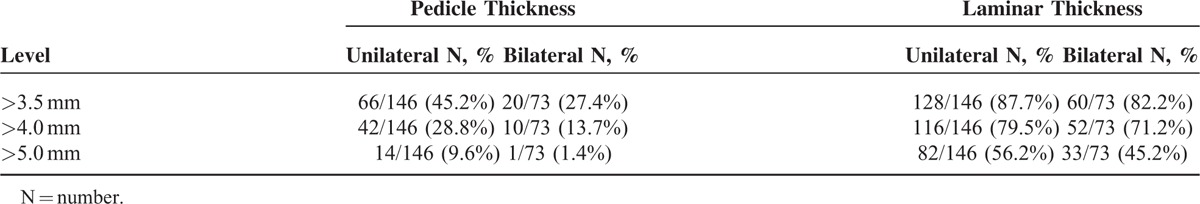
Frequency Distribution of the C2 Pedicles and Laminars at Different Levels of Thickness in the OA Group (Patients N = 73)

The mean thickness, length, and angle of the C2 lamina was 5.4 mm, 30.7 mm, and 48.9° in the OA group, respectively, with corresponding values of 6.3 mm, 32.4 mm, and 53.9° in the control group. In OA group, 12.3% of the laminas were observed to have a thickness less than 3.5 mm, whereas there were no laminar thickness values less than 3.5 mm in the control group. With regard to the frequency distribution of laminar thickness, 79.5% of the laminas were thicker than 4 mm, and 56.2% were thicker than 5 mm. Moreover, 71.2% of the bilateral laminas had thickness more than 4 mm, and 45.2% had the thickness more than 5 mm (Table 3).

The mean thickness and length of the C2 lateral mass was 8.2 and 9.3 mm in the OA group, respectively, and 9.9 and 11.6 mm in the control group, respectively. In the control group, 40% of the laminas were observed to have a length bigger than 12 mm, whereas there were no lengths of the C2 lateral mass values bigger than 12 mm in the OA group.

## DISCUSSION

The present study revealed that C2 in patients with OA often had smaller pedicle, lamina, and lateral mass in comparison with the normal people, which can lead to relatively difficult and technically demanding intervention for spinal fusion involving the craniovertebral junction. Nearly 3 quarters of our patients (53/73) have at least 1 of their pedicles less than 3.5 mm in thickness (Table 3), where the use of a 3.5 mm pedicle screw must be abandoned. In contrast, according to the measurements, the use of C2 laminar screw should not an issue for the vast majority of patients as far as the dimension is concerned. However, the lateral mass screw fixation technique in C2 does not seem to be feasible for the patients studied (mean lateral mass lengths 9.3 mm, range from 4.2 to 11.9 mm) because screws measuring 12 mm in length (the shortest screw available in our country) are required for the fixation technique. Therefore, C2 laminar screw provides a reliable alternative fixation method for OA patients when the other stabilization techniques cannot be used.

In normal circumstances, the bony space of the C1 lateral mass is sufficient to tolerate a screw with 3.5-mm diameter for fixation safely.^20–22^ However, in patients with OA, C1 lateral mass screw placement is relatively difficult and dangerous. OA is almost always associated with basilar invagination or atlantoaxial dislocation, and the C1 lateral mass is usually situated deeper and often covered by the occipital bone and C2. Thus, it is also difficult to expose the lateral mass for screw insertion.^23^ Furthermore, the small bony volume of C1 in the setting of OA means more danger for placing a screw. It is also observed that OA is sometimes accompanied by a VA anomaly. Jian et al^14^ demonstrated that in 20% of the patients with OA the VAs coursed beneath the C1 posterior arch and obstructed the proper posterior exposure of the C1 lateral mass and screw insertion. Previous studies also reported an abnormal course of the VA, running between elements of atlas and occiput despite their fusion or a persistent first intersegmental artery and VA fenestration, which make the surgical dissection and mobilization extremely difficult.^24–26^

Therefore, practically the C2 level is often an important alternative option for screw placement. However, our clinical observation indicates that the OA patients usually had smaller size of C2 pedicles than general population. Definitely, OA is a congenital disease with the possibility of multiple anomalous bony anatomies and the data of measurements of C2 from general population may not apply to patients with OA.^24,27^ Many techniques have been established for the atlantoaxial fixation or occipitocervical arthrodesis, which including the Magerl, Harms, and Wright techniques^28–31^ refer to a screw anchored at different locations of C2 vertebra. Therefore, it is of great significance to evaluate the morphometric features of C2 for screw placement in OA to guide the safe use of those fixation techniques in this specific population.

In 2014, Aoyama et al^32^ evaluate the radiographic measurement of C2 pedicles and laminas using multiplanar CT measurements for anchor screw placement in patients with C1 assimilation. This report included only 7 patients, whereas that of the present study included 73 patients. Moreover, we analyzed the length and angle of the pedicle, lamina, and lateral mass of C2, which was not done in the Aoyama et al^32^ study.

Our measurements showed that the mean C2 pedicle thickness in OA was 3.5 mm and significantly smaller than in the control group (mean 6.4 mm), and also less than that in the Aoyama et al^32^ study (mean 5.2 mm). Smith et al^33^ reported the mean pedicle thickness was 5.8 mm in normal people, which was close to the value in our study and yet far smaller than that in the Aoyama et al^32^ result (mean 7.2 mm). With pedicle thickness of 4 mm, approximately 13.7% of patients may receive pedicle screw fixation at C2 (Table 3). However, based on the previous experience, a minimum pedicle width 5.0 mm in diameter is recommended for a 3.5-mm screw placement, reserving a safety zone 0.5 to 0.75 mm from the cortex.^33,34^ Based on this requirement, the percentages dropped to only 1.4% of our patients who may receive pedicle screw fixation at C2 levels. According to Aoyama et al^32^ results, there were no pedicle thickness less than 5 mm in the right side, and yet in our study 87.7% and 93.2% of pedicle thicknesses in the right and left side, respectively, were smaller than 5 mm. Smith et at^33^ demonstrated the mean pedicle angle was 43.9°, which was close to the normal population (mean 39.5°) and bigger the angle in the patients with OA (mean 36.9°) in our study.

There is consensus that C2 is anatomically more suitable for translaminar screws fixation due to its big size of the spinous process. C2 translaminar screws fixation was indicated for salvage of failed C2 pedicle screw placement and fixation in patients with small C2 pedicles or a VA anomaly. No VA injuries were reported and even no reports exist of a compromised C2 foramen transversarium following placement of C2 translaminar screws.^35,36^ However, our experience indicates that the VA follows an anomalous course with a smaller size of C2 may also be seen in patients with OA. This pathological condition necessitate the screw placement should be more cautious than usual to avoid VA injury. Aoyama et al^32^ reported that there were no laminar thicknesses less than 3.5 mm and even 4.0 mm in the patients group. This finding was not supported by our study because there were 87.7% and 79.5% of the laminar thicknesses bigger than 3.5 and 4.0 mm, respectively, although the mean laminar thicknesses were similar in both studies. Moreover, in the normal population, the laminar thickness in our group (mean 6.3 mm) was also similar with the Aoyama et al^32^ result (mean 6.5 mm). Assuming a safety zone surrounding the translaminar screw, a minimum 5-mm laminar thickness is usually recomended.^7^ In the present study, according to the above recommendation, the translaminar screws technique is potentially feasible in 45.2% of the patients at C2. It was reported that the C2 lamina could be tolerated the mean maximum screw length was 32 mm,^37^ which is close to the dimension in our control group (mean 32.4 mm). In addition, measurements in the present study demonstrated that the C2 lamina length in the OA group (mean 30.7 mm) was very close to that in the Aoyama et al^32^ report (mean 30.4 mm) and remarkably smaller than that in the both control groups. Based on CT measurement, Yusof and Shamsi^38^ reported that the mean laminar angle was 48.8°, which was similar to the value in our OA group (mean 48.8°) and smaller than that in the control group (mean 53.9°).

Some limitations of this study must be acknowledged. The measurements were performed using radiograph images rather than directly on cadaveric specimens. Nevertheless, previous reports have demonstrated that measurements obtained from CT significantly correlated with the actual anatomic measurements of C2, and have established the guidelines for pedicle or laminar screw placement.^39–41^ In addition, the sample size for radiographic measurements was small and may have contributed to the variation of the measured values. However, as the prevalence of occipitalization in the general population ranges only from 0.08% to 2.76%,^5–7^ it is difficult to obtain a large series of patients with occipitalization for anatomic analysis.

## CONCLUSION

The variable anatomy in patients with OA needs to be taken into account when performing spinal stabilization as the C2 bony architectures are significantly smaller than the general population. In summary, we found 55% of the OA patients with anatomy that would preclude 3.5-mm pedicle screw fixation. The translaminar screw is more anatomically suitable than pedicle screw for C2 fixation in OA patients. Therefore, the appropriate anchor screw type for C2 fixation should be determined based on careful preoperative imaging and thorough consideration. The reconstructive CT is an excellent and important method to reveal the anatomy of the C2 segment prior to surgery.
